# Strengthening the US Medical Countermeasure Enterprise for Biological Threats

**DOI:** 10.1089/hs.2016.0119

**Published:** 2017-02-01

**Authors:** Sanjana Ravi, Amesh A. Adalja

Biological threats, which range from naturally occurring outbreaks of infectious disease to intentional acts of bioterrorism, pose unique dangers to public health and national security in the United States. Medical countermeasures (MCMs)—drugs, vaccines, diagnostic tests, and other therapies and devices—rank among the most important assets in the nation's arsenal against dangerous pathogens, as well as chemical, radiological, and nuclear threats. Communities with access to vital MCMs are better equipped to detect both accidental and deliberately caused outbreaks quickly, immunize susceptible populations, and treat infected patients, thereby alleviating the consequences associated with infectious diseases: illness and death, economic losses, societal unrest, and strained public health and healthcare delivery systems.

The United States relies on the Public Health Emergency Medical Countermeasures Enterprise (PHEMCE) to develop, approve, purchase, and distribute the MCMs needed to mitigate biological threats and their impacts on health and security. PHEMCE, which is led by the Department of Health and Human Services' (HHS) Office of the Assistant Secretary for Preparedness and Response (ASPR), includes several HHS partners, including the Centers for Disease Control and Prevention (CDC), the Food and Drug Administration (FDA), and the National Institutes of Health (NIH). Other interagency partners include the Department of Defense (DoD), the Department of Veterans Affairs (VA), the Department of Homeland Security (DHS), and the US Department of Agriculture (USDA). Among these entities, NIH (which includes the National Institute of Allergy and Infectious Diseases) is a driving force for early-stage biomedical research on infectious disease prevention and treatment. DoD operates a Medical Countermeasure Systems office that leads efforts to develop and field MCMs for military forces. Finally, the Biomedical Advanced Research and Development Authority (BARDA), part of ASPR, plays a key role in developing and procuring MCMs against a range of biological threats.

Several policy frameworks and pieces of legislation also govern US efforts in MCM acquisition, regulation, and distribution. Notable among these is the Project BioShield Act of 2004, which was created to fund development and government procurement of novel MCMs against traditional biological weapons threats such as botulism, anthrax, and smallpox. The FY2016 budget for HHS's Public Health and Social Services Emergency Fund included $646 million to support Project BioShield activities. The Pandemic and All-Hazards Preparedness Reauthorization Act (PAHPRA), enacted in March 2013, authorized further funding for MCM purchasing and advanced MCM research and development efforts through 2018. Additionally, Homeland Security Presidential Directives 10, 18, and 21 (Biodefense for the 21st Century, Medical Countermeasures Against Weapons of Mass Destruction, and Public Health and Medical Preparedness, respectively) underscore the importance of a robust MCM enterprise to national and global health security.

With the support of federal policies and legislation, PHEMCE has made many critical contributions to preparedness and response efforts around biological threats. In response to the 2014 Ebola outbreak in West Africa, for example, PHEMCE partners rapidly coordinated efforts to develop and test needed vaccines, therapeutics, and diagnostics. In 2014, PHEMCE partners also procured 4 million additional doses of a next-generation smallpox vaccine, as well as 33,000 doses of a monoclonal antibody against anthrax toxin. Additionally, between 2014 and 2015, FDA approved 2 new influenza products, as well additional MCMs against anthrax, high doses of radiation, and plague.

Still, sustaining and furthering national MCM preparedness for biological threats remains a challenge for the nation. Bringing a fully licensed MCM from bench to bedside typically takes up to a decade or longer and close to a billion dollars. An evolving threat landscape further complicates efforts to prioritize MCM investments for different types of biological threats. Recent experiences with Ebola, Zika, Middle East respiratory syndrome coronavirus (MERS-CoV), and pandemic influenza have also highlighted a range of challenges: for example, that pharmaceutical manufacturers have little financial incentive to develop and produce MCMs against rare and emerging—albeit high-consequence—pathogens.

Given that most MCMs against high-priority biological threats lack viable commercial markets, there is considerable uncertainty regarding their development. As a result, MCM development for biodefense and emerging infectious diseases remains contingent upon government funding, unique incentive systems, and advance purchase agreements. Federal support for MCM research and development is a prerequisite for national security and preparedness. There are several steps the next administration could take to fortify the United States's MCM enterprise. These efforts, in turn, could enhance national readiness for biological threats, protect the public's health, and strengthen national security.

## Recommendations

➢ **The President should request continued funding for basic research and investments in new technologies that could accelerate MCM development and production.**

The President's Council of Advisors on Science and Technology (PCAST), in its most recent report on US biosecurity, recommends greater funding for basic and applied research (including regulatory science) at HHS and DoD, which would begin at an initial level of $75 million annually for 4 years and gradually increase to $250 million per year. Such support is an important precondition for successful discovery and development of MCMs for biodefense.

PCAST also recommends setting an ambitious national goal: that a vaccine for a novel biological threat should be developed, manufactured, tested, and licensed within 6 months of identifying the threat and deciding to pursue a vaccine. NIH, the biomedical research arm of the federal government, should receive increased funds specifically designated for this foundational work.

In addition to supporting basic research for biological threats, the Administration should also increase investments in new technologies (eg, platform technologies and synthetic biology–based methods of rapidly creating diagnostics, drugs, and vaccines) that could accelerate MCM development and help scale-up MCM production during a public health emergency. Funds in support of this aim should be vested in BARDA, which develops technologies for advancing MCM manufacturing, and the Defense Advanced Research Projects Agency's (DARPA) Biological Technologies Office, which executes innovative biotechnology research efforts in support of national security.

➢ **The Administration should prioritize development and procurement of MCMs to address antimicrobial resistance, including nontraditional MCMs.**

Federal funding entities and research agencies need to prioritize MCMs needed to combat an evolving array of biological threats. R&D efforts should include a focus on creating point-of-care diagnostic tools, particularly for emerging or novel infections, as well as a strong focus on addressing antimicrobial resistance, given increasing resistance to current anti-infectives. New antibiotics and antivirals are needed, but research and development beyond traditional MCMs is critical. The Administration should continue to support efforts that aim to bring nontraditional MCMs to market. These include, but are not limited to monoclonal antibodies, bacteriophages, lysins, antimicrobial peptides, and microbiome-based products. The Administration should also continue to encourage federal scientists at BARDA and NIAID to identify nontraditional antimicrobials that qualify for fast-track approval, priority review, or accelerated approval pathways under the GAIN Act.

To alleviate the regulatory burdens associated with approving new MCMs, the new Administration should also encourage Congress to increase base funding for FDA to strengthen its Medical Countermeasure Initiative. These funds would enable the agency to grow and train its product review workforce; additional supplemental funds could also support FDA's ongoing regulatory scientific research efforts. Such support, in turn, would allow relevant FDA programs and offices to mobilize MCMs through the regulatory pipeline as efficiently as possible.

➢ **HHS should design and implement new incentive structures to accelerate public- and private-sector MCM research and development efforts.**

Certain incentives could spur innovation around MCMs that target drug-resistant pathogens, agents of bioterrorism, and emerging infectious diseases, while others may pose challenges. With the enactment of the 21st Century Cures Act, FDA will be tasked with overseeing a new priority review voucher program designated for MCMs against high-priority pathogens deemed to be national security threats, as well as other chemical, radiological, and nuclear agents. While such vouchers may offer pharmaceutical developers an added incentive to create needed MCMs for countering biological threats, conducting expedited product reviews over the specified 6-month period (as opposed to the typical 10-month period) could present FDA with considerable product review burdens. Increased base funding to strengthen FDA's product review workforce, as noted previously, could increase the agency's capacity to conduct expedited reviews of MCMs through this new program.

Initiating novel MCM development is only the first hurdle the nation faces in enhancing biothreat preparedness. Advanced or late-stage development is the costliest phase of the MCM pipeline and historically has been a major bottleneck in bringing viable products to market. The Administration could help bridge these late-stage gaps by increasing biodefense-related MCM funding through PAHPRA and Project BioShield—which are intended to cover costs associated with late-stage MCM development and procurement—thereby reducing some of the risks pharmaceutical manufacturers face in bringing MCMs to market.

Building public-private biosecurity partnerships could further strengthen research and development efforts at every point in the MCM value chain. The US government should create new opportunities for private-sector pharmaceutical companies to work with DoD and NIAID to translate basic research findings into investigational therapies, diagnostics, and prophylactics. The BARDA-NIH CARB-X accelerator, aimed at developing new MCMs in the face of antimicrobial resistance, is a recent example, as is BARDA's partnership with GlaxoSmithKline to develop novel antibiotics for combating both antibiotic resistance and the threat of bioterrorism. As these MCMs mature, further collaboration between manufacturers and federal entities that specialize in advanced development—such as BARDA's Centers for Innovation in Advanced Development and Manufacturing, or DoD's Medical Countermeasure Systems office—could help ensure that they successfully transition into the appropriate commercial markets.

➢ **The Administration should strengthen protocols for testing and approving MCMs for use during public health emergencies involving biological agents.**

The United States faces challenges in verifying the safety and effectiveness of MCMs for biodefense, as many cannot be ethically tested in human subjects. The Animal Efficacy Rule allows for approval of such MCMs based on evidence from animal studies; however, researchers still lack validated animal models for several high-priority diseases. The Administration should therefore fund federal biomedical authorities to continue collaborating with partners in academia and industry to identify additional animal models for basic research on emerging and intentional biological threats.

Another challenging problem, exemplified during recent outbreaks of Ebola and Zika, is that of generating enough interpretable clinical trial data to inform regulatory decision making around MCMs during ongoing emergencies. The Administration should encourage Congress to invest additional funds in creating and strengthening the infrastructure—personnel, trial sites, recruitment mechanisms, and the like—needed to conduct clinical trials efficiently both during acute public health emergencies and amid ongoing threats such as the spread of antimicrobial-resistant pathogens.

➢ **The Administration should strengthen international MCM sharing mechanisms.**

The United States is an important donor of MCMs during international public health emergencies, but often faces a myriad of legal, logistical, and financial barriers in sharing MCMs with nations affected by biological threats. During the 2009 H1N1 influenza pandemic, for example, the US government provided nearly 1 million doses of antivirals to affected nations through the Pan American Health Organization and pledged up to 25 million additional doses to the World Health Organization. In doing so, however, US health authorities encountered considerable challenges in establishing legal conditions with recipient nations. Assisting other countries in the control and containment of significant infectious disease outbreaks is in the United States's public health and national security interest. In this vein, the Administration should identify strategies for clarifying, codifying, and strengthening international MCM sharing protocols. Given an increasingly globalized world, having stronger national capacities to mitigate biological threats outside the United States lessens the risk of those threats gaining a domestic foothold.

The threat of natural and deliberate infectious disease emergencies are ever present and, by their very nature, evolving at a rapid pace. Ensuring that a robust and innovative MCM enterprise is poised to meet these challenges is the chief means of enhancing the national and health security of the nation and its people.

